# Predicting Outcome in a Cohort of Isolated and Combined Dystonia within Probabilistic Brain Mapping

**DOI:** 10.1002/mdc3.13345

**Published:** 2021-09-24

**Authors:** Carolina Soares, Martin M. Reich, Francisca Costa, Florian Lange, Jonas Roothans, Carina Reis, Rui Vaz, Maria José Rosas, Jens Volkmann

**Affiliations:** ^1^ Neurology Department Centro Hospitalar Universitário de São João, EPE Porto Portugal; ^2^ Department of Clinic Neurosciences and Mental Health, Faculty of Medicine University of Porto Porto Portugal; ^3^ Neurology Department Julius‐Maximilians‐University Würzburg Würzburg Germany; ^4^ Department of Medical Imaging, Neuroradiology Unit, Centro Hospitalar Vila Nova de Gaia/Espinho Porto Portugal; ^5^ Neuroradiology Department Centro Hospitalar Universitário de São João Porto Portugal; ^6^ Neurosurgery Department Centro Hospitalar Universitário de São João Porto Portugal

**Keywords:** combined dystonia, deep brain stimulation, pallidal neurostimulation, probabilistic map

## Abstract

**Background:**

Probabilistic brain mapping is a promising tool to estimate the expected benefit of pallidal deep brain stimulation (GPi‐DBS) in patients with isolated dystonia (IsoD).

**Objectives:**

To investigate the role of probabilistic mapping in combined dystonia (ComD).

**Methods:**

We rendered the pallidal atlas and the volume of tissue activated (VTA) for a cohort of patients with IsoD (n = 20) and ComD (n = 10) that underwent GPi‐DBS. The VTA was correlated with clinical improvement. Afterwards, each VTA was applied on the previously published probabilistic model (Reich et al., 2019). The correlation between predicted and observed clinical benefit was studied in a linear regression model.

**Results:**

A good correlation between observed and predicted outcome was found for both patients with IsoD (n = 14) and ComD (n = 7) (r^2^ = 0.32; *P* < 0.05). In ComD, 42% of the variance in DBS response is explained by VTA‐based outcome map.

**Conclusion:**

A probabilistic model would be helpful in clinical practice to circumvent unpredictable and less impressive motor results often found in ComD.

Pallidal deep brain stimulation (GPi‐DBS) is an effective and safe therapy for isolated dystonia (IsoD).^1^, ^2^, ^3^, ^4^, ^5^ Nevertheless, 10%–20% of patients show an improvement below 25%–30%.^6^ Several factors may be accountable for outcome variability: disease duration, patient age, severity and type of dystonia, variability in electrode placement and inappropriate stimulation settings.^6^ The term “combined dystonia” (ComD) encompasses different and heterogeneous disorders with a combined phenotype of dystonia and other neurological associated features (eg, myoclonus, parkinsonism, hypotonia, chorea, or ataxia).^7^ DBS appears to be more complex and unpredictable for the treatment of ComD.^8^, ^9^, ^10^ To better align patient expectations with the risks of DBS implantation, a reliable outcome prediction model would be desirable. Such a tool, based on probabilistic mapping of the antidystonic effects of pallidal neurostimulation, was recently published.^11^ This approach used the volume of tissue activated (VTA) and correlated clinical improvement of a large cohort of 87 patients with IsoD collected from several European centres to composite a probabilistic map. A robust linear regression model was trained on these 87 patients.

Probabilistic outcome brain mapping is a promising tool to estimate the expected benefit of patients with IsoD on a single subject level. Here, we aimed to assess its applicability in a completely independent cohort including ComD.

## Methods

### Patients Selection

We retrospectively included datasets of patients with IsoD (idiopathic or genetic) and ComD (inherited or acquired) that underwent bilateral GPi‐DBS at Movement Disorders Unit in University Hospital of São João, Porto. The patients were eligible if they met the following inclusion criteria: (1) video recordings depicting patients motor state before and after surgery; (2) pre‐operative MRI; (3) post‐operative CT scan. Patients with a prior ablative surgery or major complication after GPi‐DBS were excluded.

### Surgical Procedure and Clinical Assessment

All patients were implanted with quadripolar macroelectrodes (model type 3389; Medtronic Inc.) into the GPi. The neurostimulation parameters were programmed based on clinical response testing.^12^ Demographic and clinical characteristics, as well as neurostimulation settings, were collected. The severity of dystonia was assessed before and after neurostimulation (12 and 36 months post‐operatively) through Toronto Western Spasmodic Torticollis Rating Scale (TWSTRS) and Burke‐Fahn‐Marsden Dystonia Rating Scale (BFMDRS) for subjects with cervical and generalized dystonia, respectively.^13^, ^14^ Results were normalized by calculating the percentage change in TWSTRS and BFMDRS motor subscore. Patients were categorized as “non‐responders” (<25% of motor benefit); “average” (25%–50% of motor benefit); “good‐responders” (50%–80% of motor benefit); “super‐responders” (>80% of motor benefit).

### Processing of Imaging Data

Processing of the individual imaging data and registration of the lead location have been described in detail elsewhere.^11^ Images were then linearly normalized into Montreal Neurological Institute space (ICBM 2009b NLIN asymmetric). All electrodes/VTA were associated with the percentage of motor improvement to define the area within the highest probability of good outcome in our cohort. Finally, each patients VTA was applied on the previously published VTA‐based model determining the predicted motor outcome.^11^

### Statistics

Baseline analysis was performed using descriptive statistics. Categorical variables were compared using Chi‐square test. For continuous variables, we used t‐tests, Mann–Whitney U and Wilcoxon test. The correlation between predicted and observed clinical benefit was examined using a linear regression model. Multivariate regression analysis was used to analyze the effects of clinical variables on the therapeutic outcome. *P* values <0.05 were considered statistically significant.

## Results

### Clinical Characteristics and Aggregated Analysis VTA

**FIG. 1:**
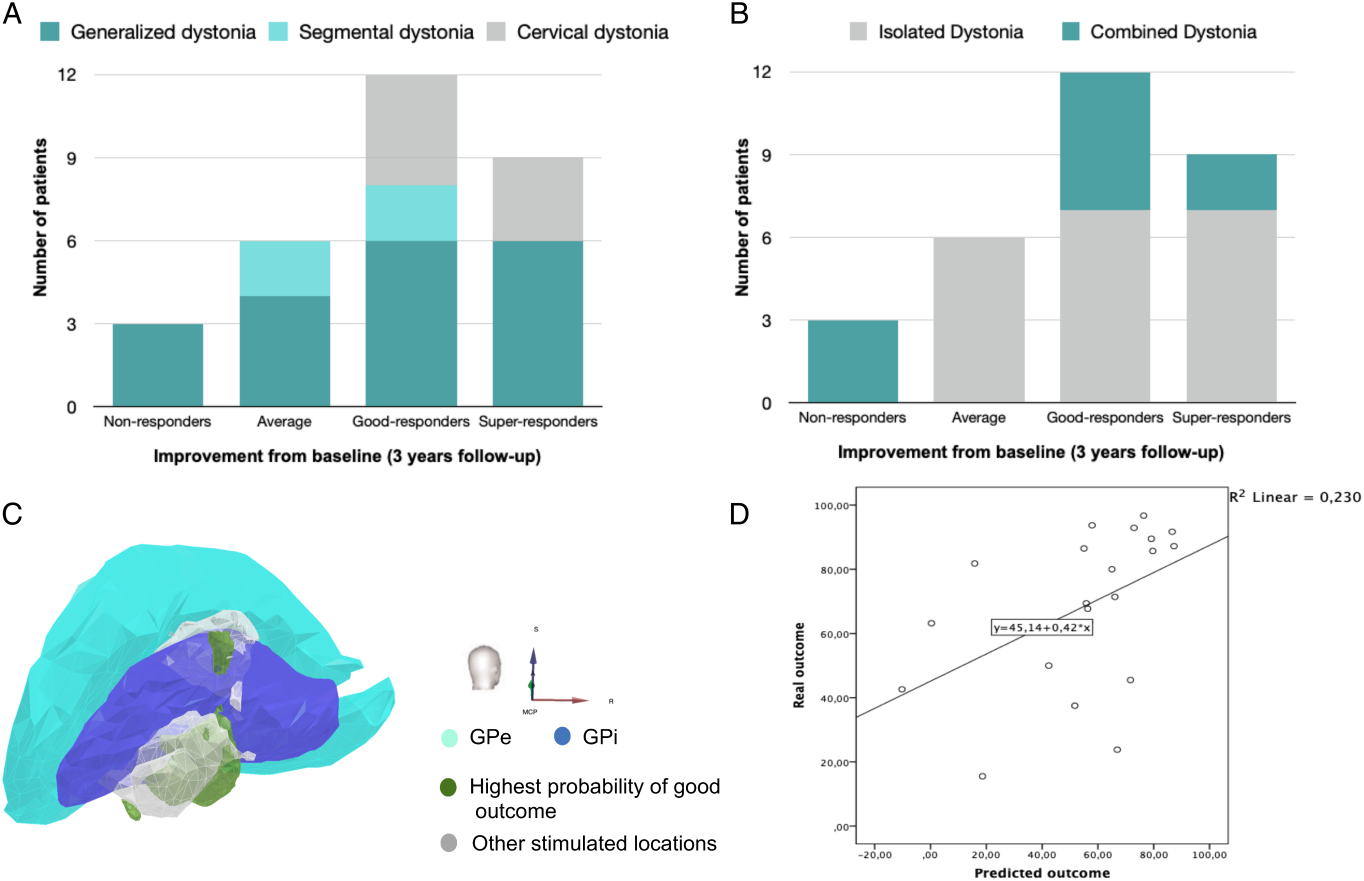
Variability of motor outcome after GPi‐DBS and analysis of VTA‐based outcome map in our cohort. Motor improvement after GPi‐DBS of all patients with dystonia (**A**). Motor improvement after GPi‐DBS of patients with IsoD and ComD (**B**). 3D‐map reconstruction depicting the aggregation of all VTA and their location into the pallidal atlas (**C**). Linear regression model analysis (**D**). GPe: Globus pallidus externa; GPi: Globus pallidus interna.

*We enrolled 30 patients with dystonia that underwent bilateral GPi‐DBS between 2005–2020*, *including* seven patients with cervical dystonia (23.3%), 19 patients with generalized dystonia (63.3%) and four patients with segmental dystonia (13.3%). Demographic and clinical characteristics are detailed in Table 1. The percentage of “non‐responders” was 10% after 3 years under chronic neurostimulation. The majority of patients had a motor improvement over 50% (Fig. 1A). The volume with the highest probability of good outcome was located within the ventroposterior GPi and adjacent subpallidal white matter (Fig. 1C).

**TABLE 1:** Demographics, clinical characteristics and motor assessment

	Cervical Dystonia	Generalized/Segmental Dystonia	P‐value
N° of patients	7	23	
Age at surgery, yrs, M(SD)	47.43 (12.79)	37.09 (10.69)	*P* = 0.226
Disease duration, yrs, M(SD)	3.86 (2.48)	18.13 (12.32)	*P* < 0.001*
Age of onset, yrs – M(SD)	43.57 (11.59)	18.96 (22.45)	*P* = 0.012^*^
Motor benefit, 1y follow‐up, %, M(SD)	70.54 (14.93)	56.85 (22.39)	*P* = 0.050
Motor benefit, 3 yrs follow‐up, %, M(SD)	78.11 (9.95)	61.94 (25.65)	*P* = 0.098
***Stimulation Parameters (1 y follow‐up)***			
Amplitude, mA, M(SD)	2.85 (0.70)	3.72 (25.02)	*P* = 0.019^*^
Pulse width, us, M(SD)	85.00 (27.39)	78.04 (25.02)	*P* = 0.469
Frequency, Hz, M(SD)	130.00 (0.00)	132.02 (22.95)	*P* = 0.371
***Stimulation Parameters (3 years follow‐up)***			
Amplitude, mA, M(SD)	3.27 (0.49	3.59 (1.01)	*P* = 0.453
Pulse width, us, M(SD)	103.33 (25.37	84.94 (36.40))	*P* = 0.032^*^
Frequency, Hz, M(SD)	130.00 (0.00)	128.87 (23.66)	*P* = 0.264

	**Isolated Dystonia**	**Combined Dystonia**	**P‐value**
N of patients	20	10	
Age at surgery, yrs, M(SD)	43.62 (18.65)	32.09 (17.97)	*P* = 0.988
Disease duration, yrs, M(SD)	13.90 (13.24)	17.73 (10.79)	*P* = 0.194
Age of onset, yrs, M(SD)	29.71 (22.58)	14.36 (20.11)	*P* = 0.034*
Gene mutation (gene)	2 out of 19 (TOR1A, THAP1)	2 out of 8 (SGCE, PANK2)	‐
Motor benefit, 1y follow‐up, %, M(SD)	60.81 (22.82)	54.84 (20.59)	*P* = 0.506
Motor benefit, 3 yrs follow‐up, %, M(SD)	69.25 (20.71)	52.73 (32.40)	*P* = 0.179
***Stimulation Parameters (1 y follow‐up)***			
Amplitude, mA, M(SD)	3.41 (0.98)	3.81 (1.27)	*P* = 0.506
Pulse width, us, M(SD)	75.36 (21.28)	94.77 (35.22)	*P* = 0.074
Frequency, Hz, M(SD)	130.00 (14.41)	136.36 (30.99)	*P* = 0.938
***Stimulation Parameters (3 years follow‐up)***			
Amplitude, mA, M(SD)	3.38 (0.80)	3.90 (1.18)	*P* = 0.228
Pulse width, us, M(SD)	77.37 (21.95)	117.75 (54.78)	*P* = 0.019*
Frequency, Hz, M(SD)	131.32 (12.12)	129.50 (34.68)	*P* = 0.982

Categorical variables were compared using Chi‐square test; Mann–Whitney U for independent variables was performed for continuous variables with non‐normal distributions; t‐tests for independent and paired samples were performed for continuous variables with normal distributions.

Abbreviations: Hz, Hertz; M, mean; us, microseconds; mA, milliampere; SD, standard deviation; y/yrs, year/years.

* Statistically significant (*P* < 0.05).

### Clinical Characteristics of IsoD Versus ComD

Our sample included 10 patients with ComD (33.3%): five patients with myoclonus‐dystonia, three patients with cerebral palsy (CP), one tardive dyskinesia and one neurodegeneration with brain iron accumulation (NBIA) (Table 1). Patients with ComD had an earlier disease onset [14.36 (20.11), *P* = 0.034] and a lower clinical improvement when compared with patients with IsoD [52.73% vs. 69.25%, *P* = 0.179]. No correlation was found between motor improvement and the age of disease onset, disease duration, age at surgery or gene mutation. All “non‐responders” had generalized ComD: two patients with CP and one patient with NBIA (Fig. 1B).

### Feasibility of VTA‐Based Outcome Map

We analyzed the feasibility of VTA‐based outcome map through a linear regression model. A total of 21 out of 30 patients were studied; nine had to be excluded in this analysis when at least one lead was not covered by the probabilistic outcome map, corresponding to six patients with IsoD and three patients with ComD.

A positive correlation between clinical measured motor score reduction and predicted motor outcomes based on probabilistic map was found (r^2^ = 0.23; *P* < 0.05) (Fig. 1D). Considering clinical and demographic variables in a multivariate analysis (age at onset, disease duration, age at surgery and baseline motor state), 32% of the observed variance in DBS response can be explained by this probabilistic model (r^2^ = 0.32; *P* < 0.05). Of interest, when only the patients with ComD were considered similar correlations were found (r^2^ = 0.42; *P* < 0.05). By contrast, predicted outcomes based on active electrode location had no correlation with the observed clinical improvement provided by GPi‐DBS (r^2^ = 0.00; *P* = 0.867) (Appendix S1).

## Discussion

We assessed the feasibility of predicting individual outcomes of GPi‐DBS in a cohort of IsoD and ComD based on a probabilistic map derived from aggregating motor outcome data and the corresponding VTA into a common anatomical reference space. To our knowledge, this is the first study to provide new insights into the feasibility of VTA‐based prediction model for pallidal neurostimulation in patients with ComD.

### Challenges of GPi‐DBS in ComD

Patients with ComD are difficult to manage in clinical practice due to clinical heterogeneity and less predictable response to GPi‐DBS.^15^ Hence, a probabilistic model might be exceptionally useful in programming and managing expectations in ComD after DBS. Several factors influence motor outcome in ComD: the occurrence of secondary complications, patient age, the complexity of pathophysiology due to an injury in developing brain, the accuracy of leads placement and therapeutic contacts with respect to the boundaries of the GPi.^16^, ^17^, ^18^, ^19^, ^20^ Previous studies reported that good results may be achieved in myoclonus‐dystonia, mainly on myoclonic component.^21^, ^22^, ^23^ A prospective multicentre pilot study evaluated the clinical efficacy of GPi‐DBS in 13 adults with CP; the response to pallidal stimulation was heterogeneous, ranging from −7.5% to 55%.^8^ The largest multicentre retrospective series of 23 patients with NBIA showed that the mean improvement in BFMDRS was 25.7% at 9–15 months; this improvement is not as great as the benefit reported in patients with primary generalized dystonia or other secondary dystonia; preoperative severity of dystonia and disease duration were predictive factors for motor improvement after surgery.^9^ In our study, the majority of patients with ComD showed an improvement that was similar to that reported in controlled studies of patients with IsoD (around 50%), but the response to GPi‐DBS was heterogeneous ranging from 15.5% to 93.7%. Although randomized controlled trials report up to 25% of non‐responders in carefully selected groups of IsoD, in our cohort 10% of patients were non‐responders and all of them had ComD. By contrast, all patients with myoclonus‐dystonia had better motor outcomes than those with other combined dystonic syndromes, suggesting that clinical phenotype may predict the response to GPi‐DBS.

### Predicting GPi‐DBS in ComD

Some questions remain regarding the optimal DBS target for dystonia in the pallidal region.^24^ Previously published approaches result in a variety of different DBS “hotspots” when used in the same dataset. The most recent models, which employed voxel‐wise statistics comparing the outcomes of each voxel against an average of other outcomes in the dataset, explained substantially greater response variance compared to classically‐described target locations.^11^, ^24^, ^25^, ^26^, ^27^ These voxel‐wise models provide the highest accuracy and predictive capabilities between detected and predefined outcome maps. Furthermore, these models explain large amounts of variance during the out‐of‐sample prediction analysis, highlighting their potential use to refine DBS delivery and in future applications like computer‐guided DBS programming.

In our study, the area with the highest probability of good motor outcome was the ventroposterior GPi and adjacent subpallidal white matter both for patients with ComD and IsoD, in line with the literature.^8^, ^11^, ^12^ This region is known to represent the sensorimotor territory, giving rise to projections to the basal ganglia recipient part of the motor thalamus.^28^ Overall, a good correlation between model predicted and clinical observed motor improvement was found, providing further evidence that variable clinical outcomes may to a relevant degree be explained by the exact location and extent of the stimulation volume within the pallidal region and adjacent white matter. The VTA‐based outcome map explained 32% of variance in our heterogenous cohort of mixed dystonia types. Notably, when only the patients with ComD were considered similar correlations were found. These findings align with a prior multicentre study of probabilistic mapping data on a large cohort of 105 patients with IsoD to resolve the optimal stimulation volume within the pallidal region.^11^ Patients with myoclonus‐dystonia showed a similar improvement in dystonic and myoclonic symptoms after GPi‐DBS, as previously described, showing that our prediction algorithm could be useful both in BFMDRS and UMRS forecast (Appendix S1)^29^. This probabilistic model would be arguably helpful in the subgroup of non‐responders. We were able to include two non‐responders in the VTA‐based outcome map analysis. The probabilistic model correctly identified the clinical motor improvement in the patient with CP (prediction = 18.6%; measured motor improvement = 15.5%), but it failed to predict motor improvement in the patient with NBIA (prediction = 66.9%; measured motor improvement = 23.8%). In this particular case, we believe that other variables are strongly implicated in the final motor outcome, namely the longer disease duration until surgery (13 years) and the presence of fixed skeletal deformities. Our cohort also included three patients with secondary ComD who presented striatopallidal lesions on brain MRI. These patients improved less than 25% after GPi‐DBS, suggesting that preservation of the cytoarchitecture of the DBS targets may be required to drive the neuronal activity responsible for the treatment.^30^, ^31^ The effect of neurostimulation depends on the relative proportions of tissue in the GPi that are stimulated, particularly when the basal ganglia network has been modified by perinatal injury to the immature brain. Moreover, the effect of DBS might be hindered by slight morphological changes that are caused by ischaemic or anoxic injury to the developing brain. Nevertheless, the effects of GPi‐DBS in different subtypes of ComD and the optimal stimulation volume within the pallidal region need to be addressed in future studies with larger cohorts to further define the VTA‐based outcome prediction model in this heterogeneous group.

### Limitations

Some limitations are worth highlighting, namely the small sample size and heterogeneity of our cohort. The BFMDRS and TWSTRS, although widely used to rate dystonia severity, does not consider the complexity of the movement disorders in ComD, such as superimposed myoclonus, choreoathetosis or associated neurological symptoms. Moreover, despite VTA‐based model correctly identified patients with a predicted good outcome whose VTA did not overlap with the area of the highest expected motor benefit, the prediction is infeasible if the leads do not fall onto the model. A general limitation of current approaches to DBS mapping is that outcome prediction is only possible for volumes of the reference space which are covered by enough stimulation data. Connectome based approaches could be helpful in the future.^32^

## Conclusions

This study fosters the utility of VTA‐based prediction model in clinical practice both for planning and programming GPi‐DBS in ComD on a single subject level. It could also be useful to identify patients whose DBS potential is significantly higher than the clinically measured benefit, highlighting the potential applicability for computer‐assisted reprogramming. A clinical approach entirely based on a probabilistic model would possibly be insufficient, since VTA‐based outcome map explains less than 50% of the variability in DBS response. One the other hand, the potential applicability of the probabilistic map in the optimization of DBS programming, especially in highly complex cases, could have a relevant impact on clinical practice. Future research focused on disease related factors underpinning motor outcome variability after surgery is needed in the group of ComD.

## Author Roles

(1) Research project: A. Conception, B. Organization, C. Execution; (2) Statistical Analysis: A. Design, B. Execution, C. Review and Critique; (3) Manuscript: A. Writing of the first draft, B. Review and Critique.

CS: 1A, 1B, 1C, 2B, 2C, 3A

MMR: 1A, 1B, 1C, 2C, 3B

FC: 1C, 2C, 3B

FL: 1C, 2C, 3B

JR: 2A, 2C, 3B

CR: 1C, 2C, 3B

RV: 2C, 3B

MJR: 1A, 2C, 3B

JV: 1A, 1B, 1C, 2C, 3B

## Disclosures

### Ethical Compliance Statement

This study was carried out with the approval of the ethics committee of each participating centre and in accordance with the Declaration of Helsinki. Informed consent was obtained for each patient. We confirm that we have read the Journal's position on issues involved in ethical publication and affirm that this work is consistent with those guidelines.

### Funding Sources and Conflicts of Interest

Dr. Martin M. Reich: supported by the German Research Foundation (DFG, Project‐ID 424778381, TRR 295); this publication was supported by the Open Access Publication Fund of the University of Wuerzburg. Dr. Jens Volkmann: supported by the German Research Foundation (DFG, Project‐ID 424778381, TRR 295); this publication was supported by the Open Access Publication Fund of the University of Wuerzburg.

### Financial Disclosures for the Previous 12 Months

Dr. Martin M. Reich: consultancy and speaking fees from Boston Scientific, Medtronic and grants from Boston Scientific and Medtronic, all unrelated to the scope of this study. Dr. Florian Lange: grants from Boston Scientific unrelated to the scope of this study. Dr. Jens Volkmann: consultancy and speaking fees from Boston Scientific, Medtronic, Newronika and grants from Boston Scientific and Medtronic, all unrelated to the scope of this study. All other authors have no disclosures to report.

## Supporting information


**Appendix** **S1:** Linear regression analysis based on VTA‐atlas model and on active electrode location of patients with IsoD and ComD.
**Table S2:** Patients with myoclonus‐dystonia included in our cohort.
