# Supplementary material for: Predicting Outcome in a Cohort of Isolated and Combined Dystonia within Probabilistic Brain Mapping
Source: Mov Disord Clin Pract. 2021 Sep 24;8(8):1234–9. doi: 10.1002/mdc3.13345 (PMC8564825; doi:10.1002/mdc3.13345)
Supplement: Supplementary file 1 — Appendix S1: Linear regression analysis based on VTA‐atlas model and on active electrode location of patients with IsoD and ComD. Table S2: Patients with myoclonus‐dystonia included in our cohort. [file MDC3-8-1234-s001.docx]

**Supplementary file**

1. **Linear regression analysis based on VTA-atlas model**
   1. **Patients with isolated and combined dystonia (n=21)**


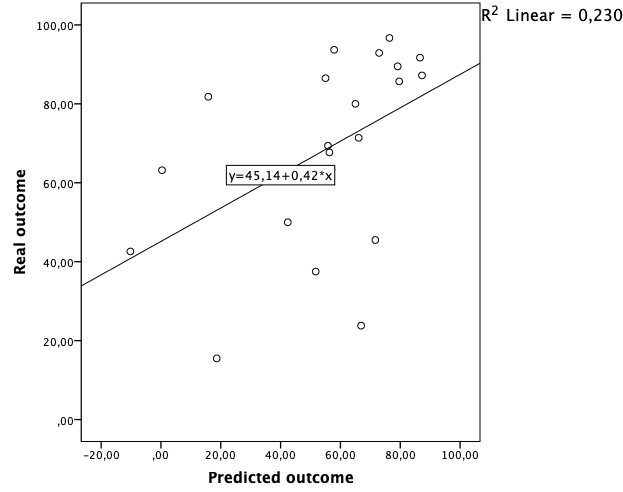


***p* < 0.05**

When adjusting for disease duration, age at surgery and score at baseline, r^2^ increased to 0.323.

|  | ***β (SE)*** | ***p-value*** |
| --- | --- | --- |
| β_0_ | 34.08 (22.06) | p=0.142 |
| Prediction | 0.41 (0.18) | p=0.042* |
| Disease duration | 0.12 (0.38) | p=0.758 |
| Age at surgery | 0.36 (0.28) | p=0.212 |
| Score at baseline | -0.16 (0.23) | p=0.478 |

Dependent variable: observed motor improvement at 3-years follow-up; r^2^=0.323; *p<0.05; residuals showed signs of normal distribution; tolerance (<4) and VIF (>0.10) showed no evidence for multicollinearity; variances homoscedasticity was observed on predicted values vs standardized residuals graph.

- 1. **Patients with combined dystonia (n=7)**


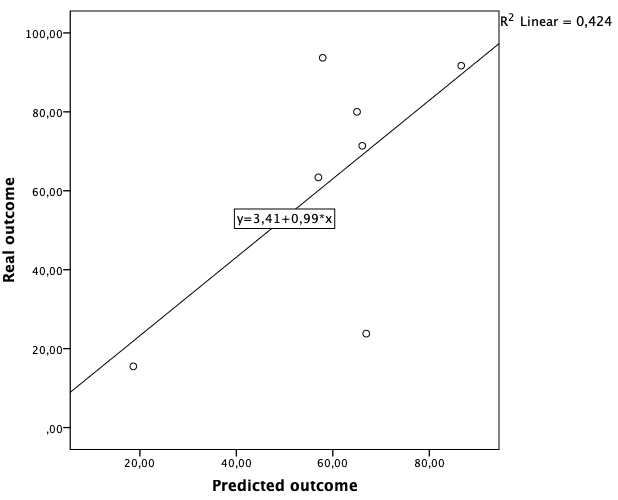


***p*=0.048**

1. **Linear regression analysis based on active electrode location (weighted average of improvement scores of its surrounding active contacts)**


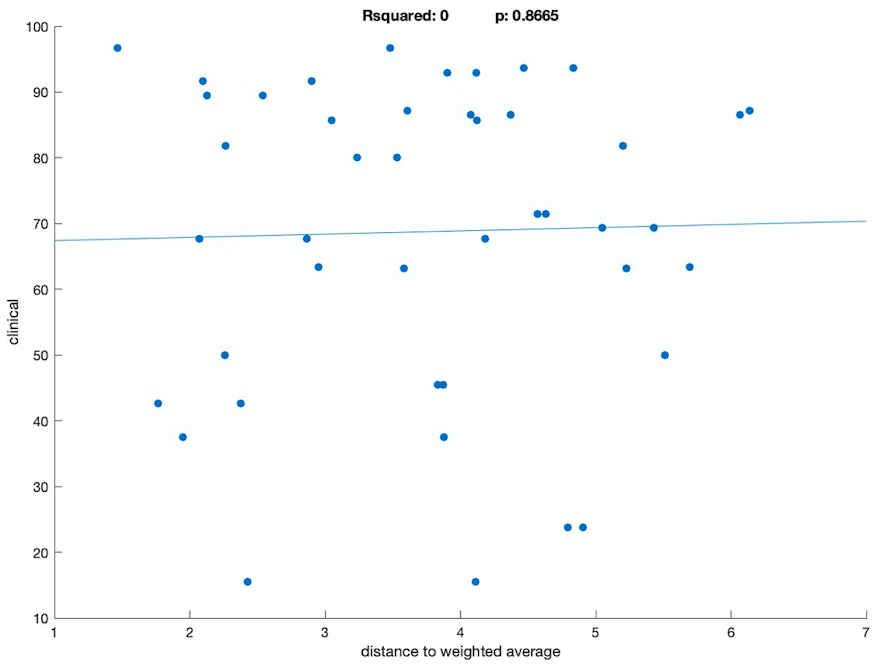


| **Table 2.** Patients with myoclonus-dystonia included in our cohort. | | | | |
| --- | --- | --- | --- | --- |
|  | Etiology | Improvement in BFMDRS (%)^§^ | Improvement in UMRS (%)^§^ | Prediction |
| Patient 1 | ε-sarcoglycane gene point mutation in exon 2 (c.158C>D p.Ser53X) | 69% | 78% | * |
| Patient 2 | Idiopathic myoclonus-dystonia syndrome | 54% | 60% | 57% |
| Patient 3 | Idiopathic myoclonus-dystonia syndrome | 70% | 79% | * |
| Patient 4 | Idiopathic myoclonus-dystonia syndrome | 43% | 54% | 66% |
| Patient 5 | Idiopathic myoclonus-dystonia syndrome | 91% | 86% | 86% |
| *Excluded from VTA-based outcome final analysis. ^§^12-months follow-up. | | | | |
